# Insights on Infiltrative and Restrictive Cardiomyopathies

**DOI:** 10.14797/mdcvj.1098

**Published:** 2022-03-14

**Authors:** Barry Trachtenberg, Mahwash Kassi

**Affiliations:** 1 Houston Methodist DeBakey Heart & Vascular Center, Methodist J.C. Walter Jr Transplant Center, Houston Methodist, Houston, Texas, US

**Keywords:** cardiomyopathy, amyloidosis, sarcoidosis, restrictive cardiomyopathy, transthyretin amyloid cardiomyopathy, granulomatous myocarditis

For years, restrictive cardiomyopathies have remained poorly understood and resulted in significant morbidity and mortality. These cardiomyopathies are often difficult to diagnose and can be considered orphan diseases that often lack definitive therapies. In the last several years, however, exciting innovation and treatment, particularly in the field of amyloidosis, has spiked new interest and hope for patients and families affected by these diseases.

In this issue, we aim to provide a comprehensive update to our readers regarding restrictive cardiomyopathies and also focus on two of the most common restrictive cardiomyopathies, amyloidosis and sarcoidosis. This issue is of special interest to us as guest editors because we both treat patients with these specific conditions, and we appreciate the opportunity to share answers to many clinically relevant questions through the reviews written by experts in the field.

The terminology and classification of restrictive (and infiltrative) cardiomyopathies often leads to much confusion. In the introductory article, Drs. Imad Hussain, Smitha Narayana Gowda, and Hyeon-Ju Ali abate the confusion in their broad overview of the definition and classification of restrictive cardiomyopathy (RCM), defined as a heterogeneous group of diseases that cause increased myocardial stiffness leading to impaired ventricular relaxation and severe diastolic dysfunction. While it is the least common type of cardiomyopathy, it can be a diagnostic challenge given varied pathogenesis, clinical presentation, and diagnostic evaluation. In this review, they provide an overview of the rarer etiologies of RCM (excluding amyloidosis and sarcoidosis) and their diagnostic and treatment approaches.

The next reviews focus on amyloidosis. Dr. Daniel Judge and colleagues provide a comprehensive overview of the epidemiology, genetics, and prognosis of transthyretin amyloid cardiomyopathy (ATTR-CM). This is an underdiagnosed disease characterized by pathologic accumulation of extracellular protein arising from unstable TTR tetramers. The authors break down the two distinct forms of ATTR-CM, hereditary and wild-type, and summarize the most common genotypes and their phenotypic heterogeneity. In addition, they give an overview of high-risk clinical features and factors that help predict prognosis.

Following the ATTR-CM discussion, Dr. Kelty Baker reviews the epidemiology of light chain amyloidosis, the staging systems, and the concomitant prognostication that is critical in determining the appropriate treatment.

Dr. Ahmad Masri and colleagues then review the clinical, imaging, and electrocardiographic clues that should raise suspicion for cardiac amyloidosis. They provide a simplified diagnostic workup algorithm to ensure an accurate diagnosis. The evolution of the noninvasive diagnosis of cardiac amyloidosis has significantly influenced our understanding of disease prevalence, presentations, and outcomes. However, clinical recognition of clues and red flags remains the most important factor in advancing the care of patients with cardiac amyloidosis.

Multimodality imaging is becoming the crux of recognizing and diagnosing cardiac amyloidosis. Dr. Mouaz Al-Mallah and colleagues highlight the role of several known and innovative cardiac imaging techniques—including echocardiography, cardiac magnetic imaging, and nuclear imaging—that aid in the evaluation of cardiac amyloidosis.

Treatment options used to be limited; however, the last decade has seen significant advances in disease-modifying therapies. Drs. Jignesh Patel and Lily Stern provide an excellent overview of current and experimental treatments for both light chain and ATTR amyloidosis.

After these excellent review articles that keep us up to date on present diagnosis and treatment of cardiac amyloidosis, Dr. Barry Trachtenberg explores where the field is heading and what the future might hold for therapeutics and diagnostics. This topic includes the goal to develop anti-fibril therapies that will be safe and effective at removing deposited amyloid fibrils and restoring organs to their pre-amyloid state, as well as the potential for genetic editing to cure variant ATTR even before patients develop clinical signs of the disease.

While several landmark studies have been done in amyloidosis, cardiac sarcoidosis is an emerging landscape for well-designed clinical trials. Several areas remain unexplored, particularly in the pathogenesis, genetics, newer imaging modalities, biomarkers, and treatment. Research and innovation are definitely needed in this area. The last two reviews of our special edition focus on this orphan condition.

Cardiac sarcoidosis (CS) is a widely underdiagnosed yet clinically significant form of granulomatous myocarditis associated with significant morbidity and mortality. Clinical presentation ranges from clinically silent disease to cardiomyopathy or sudden cardiac death. Diagnosis of CS remains challenging due to the lack of sensitivity of any single diagnostic method, underscoring the importance of elevated clinical suspicion and the use of multimodality imaging to guide diagnosis and treatment. In this review, Dr. Yogita Rochlani and her colleagues discuss the epidemiology, pathogenesis, clinical features, and diagnosis of this clinically evasive disease.

Sarcoidosis is a heterogeneous disease with various treatment indications, pulmonary and extrapulmonary. In this review, Drs. Ilias Papanikolaou, Emmanouil Antonakis, and Aggeliki Pandi provide a state-of-the art review on treatment indications and various agents whose use is supported by clinical trials.

We are grateful for the opportunity to compile this special edition and provide you with a current overview of restrictive cardiomyopathies in general, with an added focus on the two specific cardiomyopathies. Clearly, the last decade has been exciting, particularly in the arena of cardiac amyloidosis. We anticipate additional advances that will come with new innovations in bioinformatics, imaging modalities, and drug development.

## Editor Biographies

The editorial team of the *Methodist DeBakey Cardiovascular Journal* express our thanks to Dr. Trachtenberg and Dr. Kassi for their enthusiasm and dedication in curating this issue on infiltrative and restrictive cardiomyopathies.

## Barry Trachtenberg, MD

**Figure:**
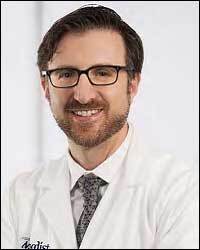


Barry Trachtenberg, MD, is a cardiologist specializing in cardio-oncology, cardiology, heart failure, and transplantation cardiology. He is board certified in internal medicine, cardiovascular disease, echocardiography, and advanced heart failure and transplant.

Dr. Trachtenberg received his BS degree from the University of Pennsylvania and attended medical school at the University of Texas-San Antonio. A native Houstonian, he completed his residency in internal medicine at Baylor College of Medicine prior to his cardiology training at the University of Miami School of Medicine.

His clinical experience includes heart failure and heart transplantation, mechanical support pumps, cardiomyopathies, cardiac amyloidosis, and cardio-oncology. He has contributed to many publications related to advanced heart failure, cardio-oncology, amyloidosis, and ventricular assist devices.

Dr. Trachtenberg is a member of the Amyloidosis Working Group of the International Cardio-Oncology Society of North America and serves on the Cardio-Oncology Council of the American College of Cardiology. He serves as the Heart Failure chair for the Texas Chapter of the American College of Cardiology. Currently, Dr. Trachtenberg is a heart failure/transplant cardiologist at Houston Cardiovascular Associates and a member of the Houston Methodist DeBakey Heart & Vascular Center and Methodist J.C. Walter Transplant Center.

## Mahwash Kassi, MD

**Figure:**
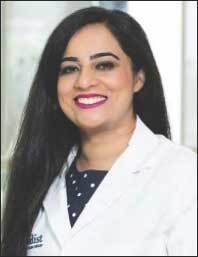


Mahwash Kassi, MD, is an advanced heart failure and transplant cardiologist at the Houston Methodist J.C. Walter Jr. Transplant Center and the Houston Methodist DeBakey Heart & Vascular Center. An assistant professor of cardiology at the Houston Methodist Academic Institute and Weill Cornell Medical College, she also is an assistant clinical member of the Houston Methodist Research Institute.

Dr. Kassi received her medical degree from Aga Khan University in Karachi, Pakistan. She completed fellowships in cardiology at Houston Methodist Hospital and in advanced heart failure and transplant at Mayo Clinic, Rochester, Minnesota, in 2017.

Her clinical interests include inherited and inflammatory cardiomyopathies, particularly cardiac sarcoidosis. She has numerous publications in peer-reviewed journals and her research interest includes computational fluid dynamic modeling in patients with left ventricular assist devices, for which she received the Texas A&M Presidential Award. In addition, Dr. Kassi is leading research efforts in cardiac sarcoidosis.

